# Development of a heptaplex PCR assay for identification of *Staphylococcus aureus* and CoNS with simultaneous detection of virulence and antibiotic resistance genes

**DOI:** 10.1186/s12866-015-0490-9

**Published:** 2015-08-05

**Authors:** Charles Emeka Okolie, Karl G. Wooldridge, David P.J. Turner, Alan Cockayne, Richard James

**Affiliations:** Centre for Healthcare Associated Infections, Centre for Biomolecular Sciences Building, The University of Nottingham, University Park, Nottingham, NG7 2RD UK; Centre for Advanced Research and Development, Landmark University, Omu-Aran, Kwara State Nigeria

**Keywords:** Differential diagnosis, *tuf* gene polymorphism, Mixed infection, Therapy-refractory

## Abstract

**Background:**

Staphylococcal toxicity and antibiotic resistance (STAAR) have been menacing public health. Although vancomycin-resistant *Staphylococcus aureus* (VRSA) is currently not as widespread as methicillin-resistant *S. aureus* (MRSA), genome evolution of MRSA into VRSA, including strains engineered within the same patient under anti-staphylococcal therapy, may build up to future public health concern. To further complicate diagnosis, infection control and anti-microbial chemotherapy, non-sterile sites such as the nares and the skin could contain both *S. aureus* and coagulase-negative staphylococci (CoNS), either of which could harbour *mecA* the gene driving staphylococcal methicillin-resistance and required for MRSA-VRSA evolution.

**Results:**

A new heptaplex PCR assay has been developed which simultaneously detects seven markers for: i) eubacteria (*16S rRNA*), ii) *Staphylococcus* genus (*tuf*), iii) *Staphylococcus aureus* (*spa*), iv) CoNS (*cns*), v) Panton-Valentine leukocidin (*pvl*), vi) methicillin resistance (*mecA*), and vii) vancomycin resistance (*vanA*). Following successful validation using 255 reference bacterial strains, applicability to analyse clinical samples was evaluated by direct amplification in spiked blood cultures (n = 89) which returned 100 % specificity, negative and positive predictive values. The new assay has LoD of 1.0x10^3^ CFU/mL for the *16S rRNA* marker and 1.0x10^4^ CFU/mL for six other markers and completes cycling in less than one hour.

**Conclusion:**

The speed, sensitivity (100 %), NPV (100 %) and PPV (100 %) suggest the new heptaplex PCR assay could be easily integrated into a routine diagnostic microbiology workflow. Detection of the *cns* marker allows for unique identification of CoNS in mono-microbial and in poly-microbial samples containing mixtures of CoNS and *S. aureus* without recourse to the conventional elimination approach which is ambiguous. In addition to the SA-CoNS differential diagnostic essence of the new assay, inclusion of *vanA* primers will allow microbiology laboratories to stay ahead of the emerging MRSA-VRSA evolution. To the best of our knowledge, the new heptaplex PCR assay is the most multiplexed among similar PCR-based assays for simultaneous detection of STAAR.

**Electronic supplementary material:**

The online version of this article (doi:10.1186/s12866-015-0490-9) contains supplementary material, which is available to authorized users.

## Background

Accumulation of genes encoding staphylococcal toxigenicity and antibiotic-resistance (STAAR) in the genus *Staphylococcus* is well documented. The evolution of STAAR factors in *Staphylococcus aureus* has resulted to the emergence of strains associated with infections recently described as life-threatening and refractory to therapy [1, 2]. Some bacterial factors are not easily detected by conventional agar-based bacteriological cultures. PCR-based assays are capable of identifying the genetic capacity for STAAR without the influence of variability in gene expression [3] and are preferred for speed and sensitivity.

Genome evolution is responsible for the emergence of strains harbouring STAAR factors some of which pose diagnostic challenges. For detection of *mecA*-encoded staphylococcal methicillin resistance, several investigators have reported multiplex PCR assays which accurately detected *mecA* gene and correlated well with the oxacillin phenotype [4, 5]. Since *S. aureus* and coagulase-negative staphylococci (CoNS) harbor the *mecA* gene in common, Huletsky and colleagues highlighted the need for a discriminatory assay capable of identifying the co-habitation of *S. aureus* and coagulase-negative staphylococci (CoNS) especially in non-sterile sites such as the nares and the skin [6]. They discriminated *S. aureus* from CoNS by detecting DNA from the staphylococcal cassette chromosome (SCC) using oligonucleotides which hybridised to the *S. aureus* open reading frame encoding unknown function (*orfX*) [6]. However, commercial assays inspired by the *orfX* oligos attracted numerous reports of assay failure because some SCC types which lacked the *mecA* gene were misidentified as MRSA [7, 8].

Basically, there are PCR-based assays for species identification of CoNS [9–11]. However, as the taxa of clinically relevant member species of the genus *Staphylococcus* continues expanding [12, 13], no species-specific assay has been capable of detecting all the species in one PCR tube. Consequently, PCR-based assays for identification of CoNS have relied on elimination based on the absence or non-detection of *S. aureus* markers such as *coa, nuc, spa* or *femA* [14]. Although the elimination approach may yield good results for mono-microbial infections, lack of a unique marker for CoNS renders such assays unreliable for identification of CoNS in poly-microbial samples containing both *S. aureus* and CoNS.

Recently the genomes of some MRSA strains evolved further. As a result of the acquisition of the *vanA* gene from enterococci vancomycin-resistant *S. aureus* (VRSA) evolved among MRSA strains [15, 16]. The recent emergence of VRSA in Portugal [17] may lead to the spread of VRSA in Europe and beyond. Furthermore, the increase in the incidence of MRSA strains belonging to the CC5 clade associated with hospital MRSA-VRSA evolution in different parts of the globe [18], including the case of the Brazilian patient whose own bloodstream vancomycin-susceptible MRSA (VS-MRSA) acquired *vanA* during antibiotic therapy and became VRSA [19] and the 13.3 % incidence of VRSA in some hospitals [20], collectively point to the hazardous capacity of this lineage, suggesting that the genetic engineering and dissemination of community associated MRSA containing *vanA* could escalate to a serious public health concern. The recent report of 46.2 % VRSA among MRSA strains isolated in chickens [21] further suggests that VRSA constitutes an impending threat to public health.

In line with the identified need for multiplex PCR assays capable of detection of multiple microbes or multiple genetic markers within the same PCR tube [22], we aimed to develop a multiplex PCR assay. Our experiments resulted in a new heptaplex PCR assay which simultaneously detected seven DNA markers for: (i) *16S rRNA* (ubiquitous bacteria), (ii) *spa* (*S. aureus*), (iii) *tuf* (the genus *Staphylococcus* spp.), (iv) cns (CoNS), (v) *pvl* (PVL virulence factor), (vi) *mecA* (methicillin resistance), and (vii) *vanA* (vancomycin resistance).

## Methods

### Bioethics and biosafety

The Research Committee, University of Nottingham, approved this study. Human-associated materials, including clinical bacterial isolates and blood cultures, were de-identified so they could not be tracked back to the patients. Many of the bacterial strains used in this study were obtained from the Network for Antibiotic Resistance in *Staphylococcus aureus* (NARSA) Strain Repository (www.narsa.net), some of which are putatively multi-resistant and/or highly virulent. An enhanced level 2 (BSL2+) suite was provided for storage and work on such potentially hazardous agents. Local strains were manipulated completely in the general (BSL2) laboratory space.

### Bacterial strains and bacteriological media used for this study

All bacteriological media and consumables used for this study were purchased from Oxoid, Basingstoke, UK. Bacterial strains (n = 255) were sub-cultured from storage (−80 °C) into brain heart infusion (BHI) broth and plated out on BHI agar. From BHI plates, a 0.5 McFarland standardised inoculum was generated for each isolate and used for gene detection and phenotypic tests. Reference type cultures (n = 53) comprising staphylococcal strains were used to validate the 7 genetic markers targeted by the new heptaplex PCR assay. Identification of the sources of the type cultures along with the numbers of strains belonging to *S. aureus* and CoNS are indicated (Additional file 1). Local clinical staphylococcal strains (n = 124) were collected from the hospitals in the Nottingham area (August 2003 - December 2004). They were previously characterized by the Queen’s Medical Centre (QMC) Nottingham NHS microbiology laboratory and de-identified by Dr. Richard Spence who used them for DNA microarrays [23]. CoNS strains (n = 31) donated by Nottingham University’s Centre for Biomolecular Sciences (CBS) researchers who previously characterized them as *S. auricularis* (2)*, S capitis* (2)*, S. caprae* (2)*, S. chromogenes* (2)*, S. cohnii* (2)*, S. epidermidis* (2), *S. hemolyticus* (1)*, S. hominis* (2), *S. hyicus* (2), *S. intermedius* (2)*, S. lugdunensis* (1), *S. saprophyticus* (3), *S. sciuri* (2), *S. simulans* (2), *S. warneri* (2) and *S. xylosus* (2) were included. Other (non-staphylococcal) bacteria (n = 47) previously characterized and de-identified by CBS researchers including strains of *Escherichia coli, Pseudomonas* spp., *Klebsiella* spp., *Aeromonas* spp., *Salmonella* spp., *Citrobacter* spp., *Proteus* spp., and Group A streptococci (GAS) were also used.

### Preparation of bacterial DNA

Preparation of bacterial cell lysates from NARSA strains followed a heating and centrifugation method reported elsewhere [24]. Bacterial DNA was obtained from spiked blood culture by Triton X-100 cell lysis method [25]. Following bacterial cell lysis step, the DNA-rich supernatant was transferred into a fresh 0.5 mL Eppendorf tube for PCR in the general (BSL2) laboratory. The lysis step was waived for local strains: bacterial colonies were directly picked and used in PCR. Similarly, broths were applied directly into PCR as previously evaluated in our laboratory and found reliable [24].

### Development and optimization of the heptaplex PCR

The *spa, pvl* and *mecA* primers were originally published by Nakagawa et al. [26]; they reliably detected *spa, pvl* and *mecA* in our laboratory [24]. The cns and 16S rRNA primers were copied from a recently published pentaplex real-time PCR assay [27]. Following other reports [28, 29], multiple alignments in clustalW (www.ebi.ac.uk/tools/clustalW) were used to design the primers targeting *tuf* and *vanA*. The greater discriminatory power of staphylococcal *tuf* over *16S rRNA* for identification of CoNS has been reported [30] and was exploited in this assay. To supply all the seven key genetic loci targeted in the new PCR, a mixed infection model comprising 3 staphylococcal strains was made up. The strains used to generate the mixed infection model were: i) vancomycin-resistant *S. aureus* strain VRS1 (*16S*, *tuf*, *spa, vanA* and *mecA*), ii) PVL-positive MSSA strain NRS157 (*16S, tuf, spa*, and *pvl*), and iii) methicillin susceptible CoNS strain *S. lugdunensis* NCTC12217 (*16S, tuf*, and *cns*). Bacterial cell lysates (10 μL), broth cultures or direct bacterial colonies were used as template for PCR in a 40 μL volume. Beginning with the five primer sets recently reported in our laboratory [27] and in a stepwise manner, the number of markers detected was scaled up to seven by adding two new primer sets targeting *tuf* and *vanA* genes. Following recent reports [31, 32], *Candida albicans* (n = 4) were used as non-bacterial negative control template while the *16S rRNA* marker for ubiquitous bacteria served as a positive control in every PCR reaction containing bacterial DNA. The PCR was performed using Eppendorf mastercycler (Eppendorf, Hamburg, Germany). The cycling conditions were copied from a recent pentaplex real-time PCR assay [27]. Briefly, an initial single cycle for 5 minutes at 94 °C was followed by 40 cycles consisting of 15 seconds at 94 °C (denaturation) and 5 seconds at 60 °C (amplification) with one final run of 30 seconds at 72 °C (final polymerase extension). PCR optimization followed recent strategies [33, 34]: hot-start PCR was performed in which Taq DNA polymerase was withheld until the cycler showed the reaction temperature was above 85 °C. The PCR mixture contained 0.500 mM of dNTPs, 10U of Taq DNA polymerase (New England Biolabs, UK) and thermopol buffer (pH 8.8 at 25 °C, 5 μL) comprising: 20 mM Tris–HCl, 10 mM (NH_4_)_2_SO_4,_ 10 mM KCl, 2 mM MgSO_4,_ 0.1 % Triton X-100. Bacterial cell lysates (10 μL) from spiked blood cultures were used as template for PCR in a 40 μL volume. PCR amplicons were resolved by electrophoresis (200 V, 3 hours) in 4 % agarose gel containing Ethidium bromide (0.5 g/L) and visualised in UV trans-illuminator (UVP, UK). PCR products were purified from agarose gels using GenElute™ (Sigma, UK). Sequencing reactions were prepared by BigDye™ protocol (AppliedBiosystems, USA) and analysed using ABI Prism 310 Genetic analyser (AppliedBiosystems, USA). Identity of PCR amplicons was confirmed by BLAST search on NCBI databases (http://blast.ncbi.nlm.nih.gov/Blast.cgi). The limit of detection (LoD) was determined by ten-fold serial dilutions performed on a 0.5 McFarland inoculum. Following isolation on BHI plates, Gram staining was performed to differentiate Gram-positive from Gram-negative bacteria. Tube coagulase test (TCT) was used to differentiate *S. aureus* from CoNS. Oxacillin salt agar screen (OSAS) containing 4 % NaCl, reported elsewhere as most reliable especially for CoNS [35, 36] was performed according to the agar dilution methods recommended by the CLSI [37]. For identification of staphylococcal vancomycin phenotype, vancomycin agar screen (VAS) was performed and interpreted according to CLSI [38]. Following CLSI recommendations [38, 39], Mueller-Hinton agar was used in disk diffusion method to screen for: (i) oxacillin-resistance using 1.0 μg oxacillin disc; and (ii) vancomycin-resistance using 6.0 μg vancomycin disc. For assay reproducibility, positive and negative characters were inferred upon the agreement of results obtained from four or more repeated gene detection and phenotypic tests performed on different days: the same 0.5McFarland inoculum prepared for PCR was used for phenotypic tests including colony counts. Aside from gene amplification from the bacterial strains performed directly on the frozen material, directly from BHI broth and directly from discrete colonies grown on BHI agar, the capacity of the new assay to detect the seven genetic markers directly from clinical samples was studied using spiked blood cultures containing bacterial strains in mono-microbial and poly-microbial models. Spiking of blood cultures followed a recent report for direct identification of staphylococci from blood culture material [40] with modifications. Briefly, the de-identified clinical blood culture bottles which have been cultured for 1 week and remained negative, derived from routine diagnostic microbiology, were spiked and then tested by PCR. Detected sequences were analysed as negative predictive values (NPV) and positive predictive values (PPV) according to recent CLSI guidelines [41].

## Results and discussion

Multiple alignments in clustalW enabled the identification of loci within the nucleotide sequences of target genes for suitable primer hybridization. Although the detection of *tuf* gene as a marker for the identification of the genus *Staphylococcus* is well documented [42], the new heptaplex PCR is the first end-point PCR-based assay which demonstrated the discriminatory power of some polymorphic regions of the staphylococcal *tuf* for unique detection of CoNS. This allows the simultaneous detection of markers specific for *S. aureus* and CoNS within the same PCR tube without recourse to the conventional elimination approach thus allowing mixtures of *S. aureus* and CoNS to be identified without cumber and ambiguity.

**Fig. 1:**
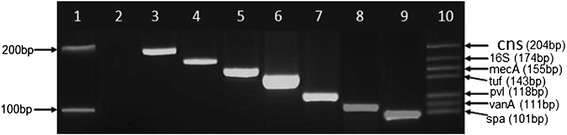
Development of the new heptaplex PCR showing the amplification of single and multiple DNA markers. Lane 1: 100 bp DNA Marker (New England Biolabs, NEB, UK) with upper band [200 bp] and lower band [100 bp], Lane 2: PCR negative control [Candida albicans], Lane 3: coagulase-negative staphylococcus marker [cns, 204 bp], Lane 4: bacterial 16S rRNA marker [16S, 174 bp], Lane 5: mecA marker [mecA, 155 bp], Lane 6: staphylococcus genus translation elongation factor marker [tuf, 143 bp], Lane 7: Panton-Valentine leukocidin marker [pvl, 118 bp], Lane 8: Vancomycin resistance marker [vanA, 111 bp], Lane 9: staphylococcal protein A marker [spa, 101 bp], Lane 10: Heptaplex PCR showing all seven markers (cns, 16S, mecA, tuf, pvl, vanA and spa) from top to bottom respectively

Bioinformatic analysis initially identified AAGACTGCACGTTCAGGCTC, a 20-letter oligonucleotide sequence as the vanA forward primer. Though the former vanA forward primer generated a vanA positive amplicon with very high specificity in a 235 bp monoplex PCR [29], it interfered with other reactions. Replacement of the interfering primer with the current vanA forward primer listed in Table 1 generated a 111 bp amplicon without interference thus allowing the simultaneous amplification of all the seven markers targeted by the new heptaplex PCR (Fig. 1).

**Table 1:** Oligonucleotide primers used in the heptaplex PCR assay

Target DNA	Amplicon size (bp)	Primer Identity	Primer sequence 5’ → 3’	Reference
cns	204	cns-1	TATCCACGAAACTTCTAAAACAACTGTTACT	[27]
		cns-2	TCTTTAGATAATACGTATACTTCAGCTTTGAATTT	
16S rRNA	174	16S-1	CTAGTAATCGCGGATCAGCAT	[27]
		16S-2	GATACGGCTACCTTGTTACGACTT	
mecA	155	mecA-1	TGGTATGTGGAAGTTAGATTGGGAT	[26]
		mecA-2	CTAATCTCATATGTGTTCCTGTATTGGC	
tuf	143	tuf-1	TACCAGCATTAGTAGTATTCTTAAACAAAGTTG	This study
		tuf-2	TGCTGAACCAGCGATTACAG	
pvl	118	pvl-1	TTACACAGTTAAATATGAAGTGAACTGGA	[26]
		pvl-2	AGCAAAAGCAATGCAATTGATG	
vanA	111	vanA-1	GCTGTGAGGTCGGTTGTG	This study
		vanA-2	GCTCGACTTCCTGATGAATACG	
spa	101	spa-1	CAGCAAACCATGCAGATGCTA	[26]
		spa-2	CGCTAATGATAATCCACCAAATACA	

**Fig. 2:**
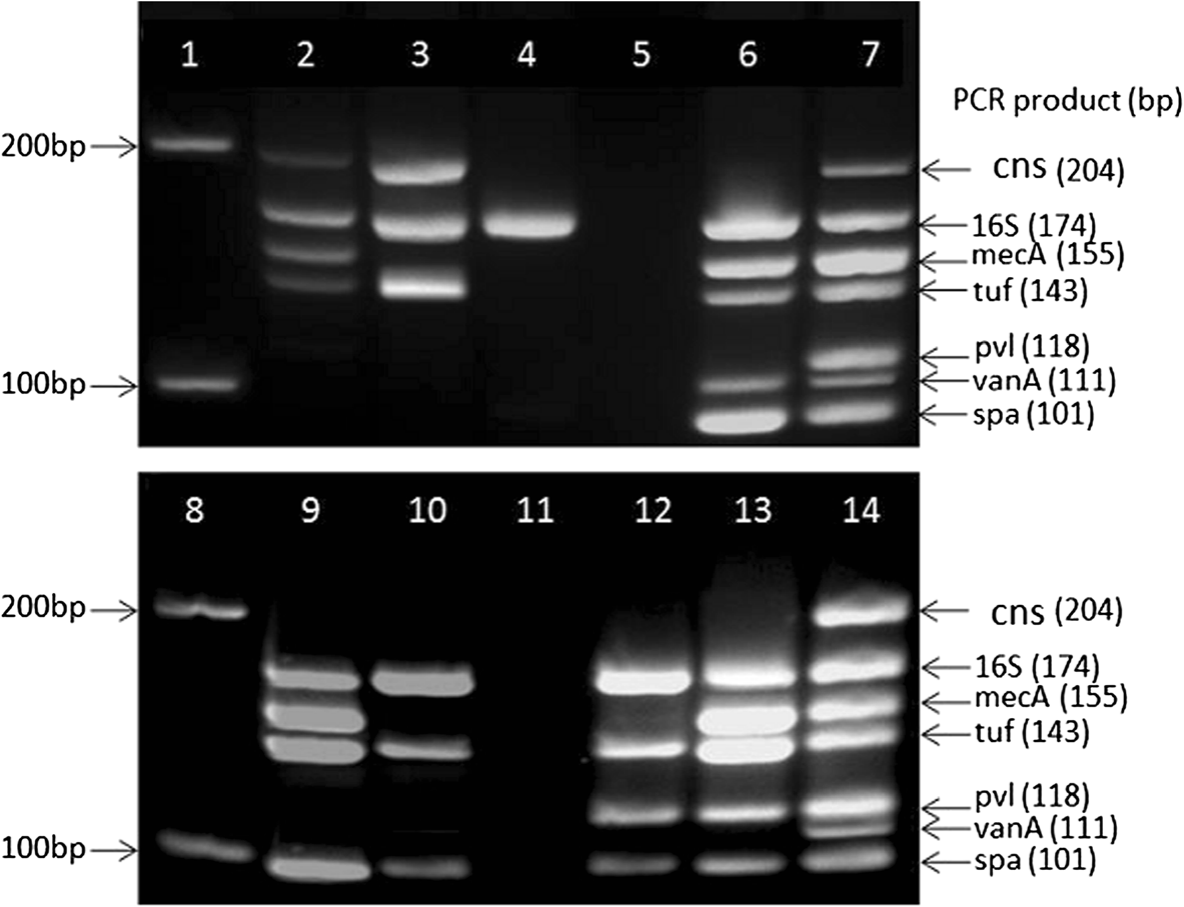
Validation of the new heptaplex PCR assay. Lanes 1 and 8: 100 bp DNA ladder (NEB, UK); Lane 2: MRCoNS strain S. epidermidis NRS8 showing the markers cns, 16S, mecA, and tuf; Lane 3: Nottingham local MSCoNS showing the markers cns, 16S, and tuf; Lane 4: Group A Streptococcus showing only the 16S marker for bacterial 16SrRNA gene; Lanes 5 and 11: PCR negative control (Candida albicans); Lane 6: VRSA strain VRS1 showing the markers 16S, mecA, tuf, vanA, and spa; Lanes 7 and 14: mixed template comprising vancomycin-resistant S. aureus strain VRS1, methicillin susceptible CoNS strain S. lugdunensis NCTC12217 and PVL-positive MSSA strain NRS157 and showing all the seven markers (cns, 16S, mecA, tuf, pvl, vanA, and spa); Lane 9: PVL-negative MRSA strain Sanger252 showing the markers 16S, mecA, tuf, and spa; Lane 10: PVL-negative MSSA strain Sanger476 showing the markers 16S, tuf, and spa; Lane 12: PVL-positive MSSA strain NRS157 showing the markers 16S, tuf, pvl, and spa; Lane13: PVL-positive S. aureus strain USA400 (MW2) showing the markers 16S, mecA, tuf, pvl, and spa

The 111bp vanA marker was amplified from all (100%) VRSA reference strains listed in Additional file 1. Upon these findings, it was inferred that useful as in silico bioinformatics work-up is in PCR primer prediction, wet experimentation is still needed to confirm the reliability of PCRs developed from such predictions. This is particularly important as very powerful bioinformatics tools are entering the oligo-design research arena, including those capable of aligning nearly a million sequences of the bacterial 16S rRNA gene [31]. Also, the lack of amplification in the negative control PCRs containing C. albicans template (Fig. 1 lane 2 and Fig. 2 lanes 5 and 11) further attests to the specificity of the new assay which is illustrated using numerous reference strains and their combinations (Fig. 2).

The optimal concentration of the oligonucleotide primers which yielded clearly detectable amplification products in the new heptaplex PCR assay were found to be 0.50 μM for cns and 0.75 μM for the other six targets.

Gene detection in spiked blood cultures, including mono- and poly-microbial cultures, showed the new assay detected all the seven targets (Additional file 2). Additional file 2 also shows that all the expected genes were correctly detected with 100 % specificity, NPV, and PPV thus enabling assignment of MSSA, PPMSSA, MRSA, PPMRSA, MSCoNS, MRCoNS, and VRSA. No PCR product was generated from the eukaryotic template, *C. albicans*, used as PCR negative control, a finding which supports its use as negative control for bacterial PCRs [31, 32].

The 111 bp *vanA* marker was not detected in the local clinical isolates. Thus the new assay corroborates the QMC phenotypic data for the local clinical staphylococci in agreement with the fact that they were uniformly susceptible to vancomycin by the VAS used to support *vanA* PCR in this study. However, one of the local strains showed discrepancy between the QMC data and gene detection by the new assay. Previously identified as methicillin susceptible *Staphylococcus epidermidis* (MSSE), the strain in question yielded PCR products for *16S, tuf, mecA* and *cns* which together characterize MRCoNS (typical of Fig. 2 lane 2). The oxacillin 1 μg disk diffusion method identified the strain as MSCoNS. Interestingly, there was growth on the 0.5 mg/L oxacillin plate while increasing concentrations inhibited the strain.

For taxonomical purposes, the relevance of species-specific PCR-based assays cannot be undermined though. However, owing to the continuing expansion in the taxa of clinically relevant member species of the genus Staphylococcus [12, 13], there is yet no species-specific PCR-based assay capable of identifying all clinically relevant CoNS in one PCR tube. This puts a limitation on species-specific PCR-based assays. To circumvent this, the new heptaplex PCR assay identifies all CoNS by detection of the cns marker. Similarly, the detection of the 16S rRNA in all (100 %) bacteria studied including staphylococcal and non-staphylococcal strains supports the use of this gene as a diagnostic marker for bacteria [43]. Though the use of higher oxacillin concentrations recommended by the CLSI [38, 39] have been reported elsewhere [44], the 0.5 mg/L oxacillin used in this study supports the recent report of low oxacillin-resistant staphylococci (MIC = 0.5 mg/L) in the UK [45]. In view of the variations in gene expression, it has been suggested that *mecA*-positive staphylococci showing low-level oxacillin-resistance should be regarded as constitutively oxacillin-resistant [3].

The new assay completes cycling within an hour which compares favourably with a recent PVL/MRSA real-time PCR assay [45]. The total turn-around-time was less than 4 hours which also compares favourably with recent end-point PCR assays [41], while the LoD was found to be 1.0x10^3^ CFU/mL (for *16S rRNA* marker) and 1.0x10^4^ CFU/mL (for the other six markers). These parameters further support the usefulness of the new heptaplex PCR assay in routine clinical diagnosis and infection control.

## Conclusion

Though the absence of *vanA* in Nottingham local clinical strains corroborates the vancomycin phenotype, the recent finding of VRSA in Portugal and the report of 46.2 % VRSA in chickens are pointers that the new heptaplex PCR assay will find relevance in routine diagnosis and infection control. Also, the detection of the cns sequence which is unique to CoNS and completely independent of *S. aureus* gene (*spa*) shows that the new assay will allow microbiology laboratories to easily identify the presence of both CoNS and *S. aureus* directly in poly-microbial specimens.

## Additional files

Additional file 1:
**Reference bacterial strains used for validation of the new heptaplex PCR assay.** (DOCX 29 kb) Additional file 2:
**Evaluation of the new heptaplex PCR assay on spiked blood cultures.** (DOCX 65 kb)
