# Supplementary material for: Development of a heptaplex PCR assay for identification of Staphylococcus aureus and CoNS with simultaneous detection of virulence and antibiotic resistance genes
Source: BMC Microbiol. 2015 Aug 5;15:157. doi: 10.1186/s12866-015-0490-9 (PMC4525735; doi:10.1186/s12866-015-0490-9)
Supplement: Additional file 1: — Reference bacterial strains used for validation of the new heptaplex PCR assay. (DOCX 29 kb) [file 12866_2015_490_MOESM1_ESM.docx]

**Additional file 1. Reference bacterial strains used for validation of the new heptaplex PCR assay**

| **Serial No.** | **Isolate** | **Species or Group** | **Source^a^** |
| --- | --- | --- | --- |
|  |  |  |  |
| 1 | NRS1 | *S. aureus* | NARSA |
| 2 | NRS102 | *S. aureus* | NARSA |
| 3 | NRS103 | *S. aureus* | NARSA |
| 4 | NRS110 | *S. aureus* | NARSA |
| 5 | NRS111 | *S. aureus* | NARSA |
| 6 | NRS112 | *S. aureus* | NARSA |
| 7 | NRS113 | *S. aureus* | NARSA |
| 8 | NRS114 | *S. aureus* | NARSA |
| 9 | NRS123 | *S. aureus* | NARSA |
| 10 | NRS13 | *S. aureus* | NARSA |
| 11 | NRS147 | *S. aureus* | NARSA |
| 12 | NRS149 | *S. aureus* | NARSA |
| 13 | NRS153 | *S. aureus* | NARSA |
| 14 | NRS157 | *S. aureus* | NARSA |
| 15 | NRS158 | *S. aureus* | NARSA |
| 16 | NRS162 | *S. aureus* | NARSA |
| 17 | NRS164 | *S. aureus* | NARSA |
| 18 | NRS165 | *S. aureus* | NARSA |
| 19 | NRS167 | *S. aureus* | NARSA |
| 20 | NRS170 | *S. aureus* | NARSA |
| 21 | NRS171 | *S. aureus* | NARSA |
| 22 | NRS172 | *S. aureus* | NARSA |
| 23 | NRS176 | *S. aureus* | NARSA |
| 24 | NRS179 | *S. aureus* | NARSA |
| 25 | NRS182 | *S. aureus* | NARSA |
| 26 | NRS185 | *S. aureus* | NARSA |
| 27 | NRS188 | *S. aureus* | NARSA |
| 28 | NRS191 | *S. aureus* | NARSA |
| 29 | NRS192 | *S. aureus* | NARSA |
| 30 | NRS194 | *S. aureus* | NARSA |
| 31 | NRS227 | *S. aureus* | NARSA |
| 32 | NRS229 | *S. aureus* | NARSA |
| 33 | NRS231 | *S. aureus* | NARSA |
| 34 | NRS233 | *S. aureus* | NARSA |
| 35 | NRS244 | *S. aureus* | NARSA |
| 36 | NRS248 | *S. aureus* | NARSA |
| 37 | NRS249 | *S. aureus* | NARSA |
| 38 | NRS255 | *S. aureus* | NARSA |
| 39 | NRS260 | *S. aureus* | NARSA |
| 40 | NRS265 | *S. aureus* | NARSA |
| 41 | NRS70 | *S. aureus* | NARSA |
| 42 | NRS71 | *S. aureus* | NARSA |
| 43 | NRS72 | *S. aureus* | NARSA |
| 44 | NRS8 | *S. epidermidis* | NARSA |
| 45 | NRS9 | *S. haemolyticus* | NARSA |
| 46 | NRS69 | *S. haemolyticus* | NARSA |
| 47 | VRS1 | *S. aureus* | NARSA |
| 48 | VRS2 | *S. aureus* | NARSA |
| 49 | VRS3 | *S. aureus* | NARSA |
| 50 | NCTC12217 | *S. lugdunensis* | NCTC |
| 51 | NCTC11042 | *S. haemolyticus* | NCTC |
| 52 | NCIMB9993 | *S. epidermidis* | NCIMB |
| 53 | NCIMB700787 | *S. capitis* | NCIMB |

^a^ Information of the gene(s) associated with the strains are available from the source web page.
