# Supplementary material for: Development of a heptaplex PCR assay for identification of Staphylococcus aureus and CoNS with simultaneous detection of virulence and antibiotic resistance genes
Source: BMC Microbiol. 2015 Aug 5;15:157. doi: 10.1186/s12866-015-0490-9 (PMC4525735; doi:10.1186/s12866-015-0490-9)
Supplement: Additional file 2: — Evaluation of the new heptaplex PCR assay on spiked blood cultures. (DOCX 65 kb) [file 12866_2015_490_MOESM2_ESM.docx]

**Additional file 2. Evaluation of the new heptaplex PCR assay on spiked blood cultures**

| **Sample number** | **Bacterial content^1^** | **Detected genes using the new heptaplex PCR assay** | | | | | | | **% Specificity** | **%NPV** | **%PPV** |
| --- | --- | --- | --- | --- | --- | --- | --- | --- | --- | --- | --- |
|  |  | cns | 16S | mecA | tuf | pvl | vanA | spa |  |  |  |
| 1 | *Candida albicans* | - | - | - | - | - | - | - | 100 | 100 | 100 |
| 2 | *S.* *epidermidis* and PN-MSSA | + | + | - | + | - | - | + | 100 | 100 | 100 |
| 3 | *S.* *lugdunensis* and PP- MRSA | + | + | + | + | + | - | + | 100 | 100 | 100 |
| 4 | *S.* *capitis* and PP-MSSA | + | + | + | + | + | - | + | 100 | 100 | 100 |
| 5 | *Saccharomyces cerevisiae* | - | - | - | - | - | - | - | 100 | 100 | 100 |
| 6 | GAS | - | + | - | - | - | - | - | 100 | 100 | 100 |
| 7 | *S. chromogenes* and GAS | + | + | - | + | - | - | - | 100 | 100 | 100 |
| 8 | *S. cohnii* and PP-MSSA | + | + | - | + | + | - | + | 100 | 100 | 100 |
| 9 | *C. albicans + S. saprophyticus* | + | + | - | + | - | - | - | 100 | 100 | 100 |
| 10 | *S. cerevisiae* + PP-MRSA | - | + | + | + | + | - | + | 100 | 100 | 100 |
| 11 | *S. hominis* and PP-MRSA | + | + | + | + | + | - | + | 100 | 100 | 100 |
| 12 | *C. albicans* + *S. cerevisiae* | - | - | - | - | - | - | - | 100 | 100 | 100 |
| 13 | *S. lugdunensis* and VRSA | + | + | + | + | - | + | + | 100 | 100 | 100 |
| 14 | *S. saprophyticus* | + | + | - | + | - | - | - | 100 | 100 | 100 |
| 15 | *S. sciuri* + PP-MRSA | + | + | + | + | + | - | + | 100 | 100 | 100 |
| 16 | *S. warneri* + *C. albicans* | + | + | - | + | - | - | - | 100 | 100 | 100 |
| 17 | *Escherichia coli* | - | + | - | - | - | - | - | 100 | 100 | 100 |
| 18 | *Pseudomonas* spp. + PP-MRSA | - | + | + | + | + | - | + | 100 | 100 | 100 |
| 19 | *Klebsiella* spp. + PP-MRSA | - | + | + | + | + | - | + | 100 | 100 | 100 |
| 20 | *Aeromonas* spp. + PP-MRSA | - | + | + | + | + | - | + | 100 | 100 | 100 |
| 21 | *Salmonella* spp. + PP-MSSA | - | + | - | + | + | - | + | 100 | 100 | 100 |
| 22 | *Citrobacter* spp. + PP-MRSA | - | + | + | + | + | - | + | 100 | 100 | 100 |
| 23 | *Proteus* spp. | - | + | - | - | - | - | - | 100 | 100 | 100 |
| 24 | GAS + *S. epidermidis* | + | + | _+_ | + | - | - | - | 100 | 100 | 100 |
| 25 | *S. caprae* + GAS | + | + | - | + | - | - | - | 100 | 100 | 100 |
| 26 | *S.* *haemolyticus* and PP-MRSA | + | + | + | + | + | - | + | 100 | 100 | 100 |
| 27 | *S.* *epidermidis* and PP-MRSA | + | + | + | + | + | - | + | 100 | 100 | 100 |
| 28 | *S.* *lugdunensis* and PP- MRSA | + | + | + | + | + | - | + | 100 | 100 | 100 |
| 29 | *S.* *xylosus* and GAS | + | + | - | + | - | - | - | 100 | 100 | 100 |
| 30 | *S. capitis* + VRSA | + | + | + | + | - | + | + | 100 | 100 | 100 |
| 31 | *S. hominis* + GAS | + | + | - | + | - | - | - | 100 | 100 | 100 |
| 32 | *Proteus spp.* + PP-MSSA | - | + | - | + | + | - | + | 100 | 100 | 100 |
| 33 | *S. cohnii* + VRSA | + | + | + | + | - | + | + | 100 | 100 | 100 |
| 34 | *S. hemolyticus* + PP-MRSA | + | + | + | + | + | - | + | 100 | 100 | 100 |
| 35 | *S. hominis* + PP-MSSA | + | + | - | + | + | - | + | 100 | 100 | 100 |
| 36 | *Klebsiella* spp. + *S. warneri* | + | + | + | + | - | - | - | 100 | 100 | 100 |
| 37 | *Aeromonas* spp. + *E. coli* | - | + | - | - | - | - | - | 100 | 100 | 100 |
| 38 | *Salmonella* spp. + PN-MSSA | - | + | - | + | - | - | + | 100 | 100 | 100 |
| 39 | *S. lugdunensis* + PP-MRSA | + | + | + | + | + | - | + | 100 | 100 | 100 |
| 40 | *E. coli* + *S. epidermidis* | + | + | _+_ | + | - | - | - | 100 | 100 | 100 |
| 41 | PP-MSSA + PN-MRSA | - | + | + | + | + | - | + | 100 | 100 | 100 |
| 42 | *S. epidermidis* + PP-MSSA | + | + | + | + | + | - | + | 100 | 100 | 100 |
| 43 | *S. hominis* + PP-MRSA | + | + | + | + | - | - | + | 100 | 100 | 100 |
| 44 | *S. sciuri* + PN-MSSA | + | + | - | + | - | - | + | 100 | 100 | 100 |
| 45 | *S. simulans +* PP-MSSA | + | + | + | + | + | - | + | 100 | 100 | 100 |
| 46 | *S. heamolyticus +*PN-MSSA | + | + | + | + | - | - | + | 100 | 100 | 100 |
| 47 | *S. heamolyticus +C. albicans* | + | + | + | + | - | - | - | 100 | 100 | 100 |
| 48 | *S. hyicus* + PP-MRSA | + | + | - | + | + | - | + | 100 | 100 | 100 |
| 49 | *S. simulans* + PN-MSSA | + | + | + | + | - | - | + | 100 | 100 | 100 |
| 50 | *S.* *epidermidis* and PP-MSSA | + | + | - | + | + | - | + | 100 | 100 | 100 |
| 51 | *S.* *lugdunensis* and PP- MRSA | + | + | + | + | + | - | + | 100 | 100 | 100 |
| 52 | Candida albicans | - | - | - | - | - | - | - | 100 | 100 | 100 |
| 53 | *C. albicans*  and PP- MRSA | - | + | + | + | + | - | + | 100 | 100 | 100 |
| 54 | *S. simulans +* PP-MSSA | + | + | + | + | + | - | + | 100 | 100 | 100 |
| 55 | *S. heamolyticus +*PN-MSSA | + | + | + | + | - | - | + | 100 | 100 | 100 |
| 56 | *S. cohnii* and PP-MRSA | + | + | + | + | + | - | + | 100 | 100 | 100 |
| 57 | *S. hyicus* + PP-MRSA | + | + | - | + | + | - | + | 100 | 100 | 100 |
| 58 | *S. simulans* + PN-MSSA | + | + | + | + | - | - | + | 100 | 100 | 100 |
| 59 | *S.* *epidermidis* and PP-MSSA | + | + | - | + | + | - | + | 100 | 100 | 100 |
| 60 | *S.* *lugdunensis* and PP- MRSA | + | + | + | + | + | - | + | 100 | 100 | 100 |
| 61 | *S. auricularis* and VRSA | + | + | + | + | - | + | + | 100 | 100 | 100 |
| 62 | *E. coli* + *S. epidermidis* | + | + | _+_ | + | - | - | - | 100 | 100 | 100 |
| 63 | PP-MSSA + PN-MRSA | - | + | + | + | + | - | + | 100 | 100 | 100 |
| 64 | *S. epidermidis* + PP-MSSA | + | + | + | + | + | - | + | 100 | 100 | 100 |
| 65 | *S. hominis* + PP-MRSA | + | + | + | + | - | - | + | 100 | 100 | 100 |
| 66 | *S. sciuri* + PN-MSSA | + | + | - | + | - | - | + | 100 | 100 | 100 |
| 67 | *S. cohnii* and PP-MRSA | + | + | + | + | + | - | + | 100 | 100 | 100 |
| 68 | *S. cervisiae +*PN-MSSA | - | + | - | + | - | - | + | 100 | 100 | 100 |
| 69 | *S. capitis* + VRSA | + | + | + | + | - | + | + | 100 | 100 | 100 |
| 70 | *S. hominis* + GAS | + | + | - | + | - | - | - | 100 | 100 | 100 |
| 71 | *Proteus spp.* + PP-MSSA | - | + | - | + | + | - | + | 100 | 100 | 100 |
| 72 | *S. cohnii* + VRSA | + | + | + | + | - | + | + | 100 | 100 | 100 |
| 73 | *S. hemolyticus* + PP-MRSA | + | + | + | + | + | - | + | 100 | 100 | 100 |
| 74 | *S. hominis* + PP-MSSA | + | + | - | + | + | - | + | 100 | 100 | 100 |
| 75 | *Klebsiella* spp. + *S. warneri* | + | + | + | + | - | - | - | 100 | 100 | 100 |
| 76 | *Aeromonas* spp. + *E. coli* | - | + | - | - | - | - | - | 100 | 100 | 100 |
| 77 | *Salmonella* spp. + PN-MSSA | - | + | - | + | - | - | + | 100 | 100 | 100 |
| 78 | *S. lugdunensis* + PP-MRSA | + | + | + | + | + | - | + | 100 | 100 | 100 |
| 79 | *S. cerevisiae* | - | - | - | - | - | - | - | 100 | 100 | 100 |
| 80 | *S. cohnii* and *C. albicans* | + | + | - | + | - | - | - | 100 | 100 | 100 |
| 81 | *S. simulans* and PN-MSSA | + | + | + | + | - | - | + | 100 | 100 | 100 |
| 82 | *S. chromogenes* and GAS | + | + | - | + | - | - | - | 100 | 100 | 100 |
| 83 | *S. cohnii* and PP-MSSA | + | + | - | + | + | - | + | 100 | 100 | 100 |
| 84 | *Candida albicans* | - | - | - | - | - | - | - | 100 | 100 | 100 |
| 85 | *S. simulans* and PN-MSSA | + | + | - | + | - | - | + | 100 | 100 | 100 |
| 86 | *S. hominis* and PP-MRSA | + | + | + | + | + | - | + | 100 | 100 | 100 |
| 87 | *C. albicans* and *E. coli* | - | + | - | - | - | - | - | 100 | 100 | 100 |
| 88 | *S. lugdunensis* and VRSA | + | + | + | + | - | + | + | 100 | 100 | 100 |
| 89 | *S. saprophyticus* | + | + | - | + | - | - | - | 100 | 100 | 100 |
| 90 | *C. albicans* | - | - | - | - | - | - | - | 100 | 100 | 100 |
| 91 | *S. hominis +*PN-MRSA | + | + | + | + | - | - | + | 100 | 100 | 100 |

^1^Based on genomic information for each strain available from the supplier or phenotypic records on the QMC databases.

^2^Nottingham clinical strains were previously identified as *S. aureus* or MRSA and *S. epidermidis* or MRSE: No information on PVL and vancomycin resistance at the time.
